# 

**Published:** 1888-12

**Authors:** 


D. A. Fraser

UFFCULME.

F. Morgan

WINKLEIGH.

J. H. Norman

### DORSETSHIRE.

BEAMINSTER.

T. P. Daniel

BRIDPORT.

G. M. Evans

CERNE ABBAS.
E. W. Kerr

DORCHESTER.
F. B. Fisher
P. W. MacDonald

PARKSTONE.

J. R. Philpots

POOLE.

J. McNicoll

PORTLAND.

G. H. Lilley

SHERBORNE.

W. H. Williams

STURMINSTER NEWTON.

J. C. Leach

THORNCOMBE.

T. A. Palm

WEYMOUTH.

R. W. Carter

WIMBORNE.

A. J. H. Crespi

### ESSEX.

SOUTHEND.
E. E. Phillips

### GLOUCESTERSHIRE.

BRISTOL.

W. R. Ackland

J. H. Parry

T. C. Parson

G. C. P. Pauli

F. Penny

J. H. Perkins

C. F. Pickering

W. E. Pountney

A. Prichard

A. W. Prichard

J. E. Prichard

A. Pring

### GLOUCESTERSHIRE *(Continued).*

BRISTOL *(continued).*

A. B. Prowse

W. Wickham

THORNBURY.
E. M. Grace
T. H. Taylor

WESTBURY-ON-TRYM.

H. Ormerod

A. W. Riddell

WINTERBOURNE.

R. Eager

WOTTON-UNDER-EDGE

D. H. Forty

### HAMPSHIRE.

BOURNEMOUTH.

D. C. Embleton

E. P. Philpots

EMSWORTH.

L. Stephens

LYMINGTON.

J. Rendall

SOUTHAMPTON.

Army Medical Staff Library

D. B. Smith

SOUTHSEA.

H. Rundle

### HEREFORDSHIRE.

HEREFORD.

W. C. Whitfeld

ROSS.

J. W. Norman

### HERTFORDSHIRE.

ST. ALBANS.

A. H. Boys

WALTHAM CROSS.

W. B. Kesteven

### KENT.

TUNBRIDGE WELLS.
C. Wilson.

### LANCASHIRE.

LIVERPOOL.

Medical Institution

POULTON-LE-FYLDE.

P. H. Day

### MIDDLESEX.

LONDON.

J. Abercombie

L. S. Beale

L. Browne

F. Cock

H. Cooper

E. East

J. F. Evans

A. P. Gould

G. S. Gregory

M. Grigor

G. Hett

G. W. Isaac

C. M. Jessop

J. Lister

W. J. Penny

M. W. H. Russell

W. J. Seward

Royal Med. and Chir. Society

G. D. Thomas

H. C. Thurston

J. E. Trask

TOTTENHAM.

W. H. Plaister

### MONMOUTHSHIRE.

ABERGAVENNY.

S. H. Steel

CAERLEON.

J. A. Morris

EBBW VALE.

J. W. Davies

F. Stockwell

BURNHAM.

A. W. C. Peskett

CHEDDAR.

R. W. Statham

CHEW MAGNA.

E. T. Hale

CLEVEDON.

T. Davis

G. F. P. Pizey

S. Skinner

CREWKERNE.

W. W. Webber

DULVERTON.

R. J. Collyns

### SOMERSETSHIRE *(Continued).*

FROME.

F. Parsons

GLASTONBURY.

J. A. Bright

ILMINSTER.

### STAFFORDSHIRE.

DARLASTON.

J. E. Gabb

STAFFORD.

J. Scott

### SURREY.

EGHAM.

W. A. Moynan

SURBITON.

J. G. Davey

### SUSSEX.

ST. LEONARD'S-ON-SEA.
W. H. Spencer

### WARWICKSHIRE.

BIRMINGHAM.
L. Tait

### WILTSHIRE.

BRADFORD-ON-AVON.

W. D. Lovell

BURBAGE.

A. E. N. Browne

CALNE.

W. S. Batten

CHIPPENHAM.

G. H. Daly

DEVIZES.

T. B. Anstie

C. Eddowes

DOWNTON.

G. W. Whiteley

FOVANT.
C. Clay

MALMESBURY.

R. Kinneir

SALISBURY.

Medical Society

C. T. Vachell

H. R. Vachell

MERTHYR TYDVIL.

J. L. W. Ward

REYNOLDSTONE.

H. V. Ellis

SWANSEA.

G. Padley

J. A. Rawlings

J. Thomas

### MONTGOMERYSHIRE.

LLANSAINTFFRAID.

T. Edwardes

### PEMBROKESHIRE.

TENBY.

B. Kendall

### Switzerland.

DAVOS PLATZ.

W. R. Huggard

### INDEX.

Abortion, Treatment of incomplete—Dr. A. E. A. Lawrence, 8.
Allingham, W.—Diseases of rectum *(Review)*, 280.
Anæsthetics—Dr. D. W. Buxton *(Review)*, 281.
Aneurism of aorta, Case of—Dr. J. M. Clarke, 130.
Aneurisms and tubercle-bacillus *(Extract)*, 212.
Annual of the universal medical sciences *(Review)*, 282.
Annual, The medical *(Review)*, 55.
Antipyrin (*Extracts*), 66, 146.
Aphasia—Dr. J. Ross *(Review)*, 281.
Arsenic, Pharmacology of (*Extract*), 214.
Asthma, Case of surgical—W. J. Penny, 41.
Athothis—T. C. Minor (*Review*), 54.
Atrophy, Case of progressive muscular—Dr. J. M. Clarke, 195.

Barker, A. E. J.—Manual of surgical operations (*Review*), 53.
Baron, Dr. B. J.—Phthisical hoarseness and aphonia, 12.
Bennett, Sir R.—Diseases of Bible (*Review*), 51.
Bible, Diseases of—Sir R. Bennett (*Review*), 51.
Bladder, Electric illumination of—E. H. Fenwick (*Review*), 203.
Bones, Diseases of—T. Jones (*Review*), 202.
Bramwell, Dr. B. — Intracranial tumours (*Review*), 283.
Breast, Diseases of — T. Bryant (*Review*), 135.
Bryant, T. — Diseases of breast (*Review*), 135.
Buxton, Dr. D. W.—Anæsthetics (*Review*), 281.

Cancer and tumour formation—W. R. Williams (*Review*), 203.
Cardiocentesis (*Extract*), 64.
Carter and W. A. Frost, R. B.—Ophthalmic surgery (*Review*), 137.
Cat-bite, Cases of inflammation from—W. J. Penny, 42.
Chancres of lip and gum—W. J. Penny, 46, 47.
Cholera, Preventive vaccination in Asiatic (*Extract*), 211.
Chorea, Case of adult—Dr. J. M. Clarke, 131.
Chorea, Optic atrophy following (*Extract*), 210.
Clarke, Dr. J. M.—Adult chorea, 131; aneurism of aorta, 130; case of progressive muscular atrophy, 195; disease of heart, 126; some spinal cases, 120.
Club-foot, Congenital (*Extract*), 68.
Coats, Drs. W. T. Gairdner and J.—Lectures to practitioners (*Review*), 203.
Cocaine as aid to diagnosis of laryngeal infiltrations (*Extract*), 150.
Contrexéville waters (*Extract*), 147.
Coryza, Atrophic (*Extract*), 298.
Crespi, A.—Droitwich and its brine baths, 1.
Crocker, Dr. H. R.—Diseases of skin (*Review*), 201.
Cross, F. R.—Notes from the seventh International Ophthalmological Congress, 215.

Dacre, J.—Intubation of larynx, 73.
Davies, Dr. D. S.—Disinfection, 83.
Death, Cause of—A. Prichard, 153.
Delivery, The hour of—Dr. J. G. Swayne, 174.
Dermatology, The British journal of (*Review*), 288.

Diagnosis, Manual of clinical—Drs. Seifert and Müller (*Review*), 56.
Disinfection—Dr. D. S. Davies, 83.
Droitwich and its brine baths—A. Crespi, 1.
Drug-action, Primary and secondary (*Extract*), 213.
Dysentery, Bacillus of epidemic (*Extract*), 145.

Ear, Diseases of—W. R. H. Stewart (*Review*), 284.
Ear, Suppuration of (*Extract*), 297.
Eczema—Dr. T. Robinson (*Review*), 198.
Electrothérapie (*Review*), 199.
Epistaxis, Cocaine in (*Extract*), 150.
Eye, Diseases of—Dr. H. R. Swanzy (*Review*), 200.

Fenwick, Dr. S.—The saliva as a test for the functional disorders of the liver (*Review*), 136.
Fenwick, E. H.—Electric illumination of bladder (*Review*), 203.
Ferris & Co.—Pocket therapeutic notes (*Review*), 139.
Frost, R. B. Carter and W. A.—Ophthalmic surgery (*Review*), 137.

Gairdner and J. Coats, Drs. W. T.—Lectures to practitioners (*Review*), 203.
Gonorrhœal infection in women—Dr. W. J. Sinclair (*Review*), 135.
Gordon, Dr. C. A.—Rabies and hydrophobia (*Review*), 137.
Gout—Dr. R. Roose (*Review*), 138.

Harris, Drs. D. Power and V. D.—Manual for the physiological laboratory (*Review*), 282.
Harsant, W. H.—Surgery of the thyroid body, 265.
Head-injuries and inflammation of lungs (*Extract*), 69.
Heart-disease, case of—Dr. J. M. Clarke, 126.
Holmes, T.—A treatise on surgery (*Review*), 276.
Hydrophobia, Rabies and—Dr. C. A. Gordon (*Review*), 137.

Inebriety—Dr. N. Kerr (*Review*), 135.
Infant feeding—Dr. J. McNaught (*Review*), 285.
Intestinal obstruction, Discussion on (*Extract*), 67.

Jamieson, Dr. W. A.—Diseases of skin (*Review*), 138.
Jollye, F. W.—Case of gouty peripheral neuritis, 28.
Jones, T.—Diseases of bones (*Review*), 20.

Kerr, Dr. N.—Inebriety (*Review*), 135.

Laffan, T.—The medical profession of the United Kingdom (*Review*), 285.
Laryngeal stenosis (*Extract*), 297.
Larynx, Intubation of—J. Dacre, 73; pachydermia verrucosa of (*Extract*), 150.
Lawrence, Dr. A. E. A.—Treatment of incomplete abortion, 8.
Lexicon, Medical—E. Longley (*Review*), 286.
Library, Catalogue of Lewis's medical (*Review*), 204.
Locomotor ataxy, Eye-troubles in (*Extract*), 210.
Longley, E.—Medical lexicon (*Review*), 286.

McNaught, Dr. J.—Infant feeding (*Review*), 285.
Medical essays—Dr. W. Squire (*Review*), 288.
Medical profession of the United Kingdom—T. Laffan; W. Rivington (*Reviews*), 285.
Medicine, Handbook of—Dr. F. T. Roberts (*Review*), 202.
Menorrhagia and hygienics—Dr. J. Meredith, 17, 102.
Meredith, Dr. J.—Menorrhagia and hygienics, 17, 102.
Minor, T. C.—Athothis (*Review*), 54.
Morbus cæruleus, Case of—Dr. P. W. Williams, 183.
Müller, Drs. Seifert and—Manual of clinical diagnosis (*Review*), 56.

Nall, Dr. S.—Aids to obstetrics (*Review*), 52.
Nasal mucous membrane, Action of caustics on (*Extract*), 149.

Nerve prostration—Dr. R. Roose (*Review*), 287.
Neuritis, Case of gouty peripheral—F. W. Jollye, 28.

Obstetrics, Aids to—Dr. S. Nall (*Review*), 52.
Ogle, Dr. J. W.—Tympanites (*Review*), 283.
Ophthalmic surgery—R. B. Carter and W. A. Frost (*Review*), 137.
Ophthalmological congress, Notes from the seventh international—F. R. Cross, 215.
Orchitis, Case of rheumatic—W. J. Penny, 49.
Otitis, Infectious (*Extract*), 70.

Pathology of intra-uterine death—Dr. W. O. Priestley (*Review*), 55.
Penny, W. J.—Out-patient cases: Patent urachus, 36; affections of the tongue, 37-40; surgical asthma, 41; inflammation from cat-bite, 42; constitutional syphilis, 45; chancres of lip and gum, 46, 47; Raynaud's disease, 48; rheumatic orchitis, 49.
Pharmacology, Elements of—Dr. O. Schmiedeberg (*Review*), 198.
Phenacetin (*Extract*), 215.
Phonetic troubles (*Extract*), 295.
Phthisical hoarseness and aphonia—Dr. B. J. Baron, 12.
Phthisis and the germ theory—Dr. R. S. Smith, 225.
Phthisis, Hæmoptysis in (*Extract*), 147.
Phthisis, Sulphurous acid in (*Extract*), 210.
Phthisis, Two cases of—Dr. R. S. Smith, 185.
Physiological laboratory, Manual for the—Drs. V. D. Harris and D. Power (*Review*), 282.
Power, Drs. V. D. Harris and D.—Manual for the physiological laboratory (*Review*), 282.
Practitioners, Lectures to—Drs. W. T. Gairdner and J. Coats (*Review*), 203.
Preparations for the sick, Notes on—57, 140, 205, 291.
Prichard, A.—Cause of death, 153.
Priestley, Dr. W. O.—Pathology of intra-uterine death, 55.

Rabies and hydrophobia—Dr. C. A. Gordon (*Review*), 137.
Raynaud's disease, Cases—W. J. Penny, 48; Dr. H. Waldo, 272.
Rectum, Diseases of—W. Allingham (*Review*), 280.
Ringworm—Dr. G. Thin (*Review*), 279.
Rivington, W.—The medical profession of the United Kingdom (*Review*), 285.
Roberts, Dr. F. T.—Handbook of medicine (*Review*), 202.
Robinson, Dr. T.—Eczema (*Review*), 198.
Roose, Dr. R.—Gout (*Review*), 138; nerve prostration (*Review*), 287.
Ross, Dr. J.—Aphasia (*Review*), 281.

Salicylate of soda (*Extract*), 146.
Saliva as a test for the functional disorders of the liver, The—Dr. S. Fenwick (*Review*), 136.
Schmiedeberg, Dr. O.—Elements of pharmacology (*Review*), 198.
Scraps, 71, 151, 223, 299.
Sea-sickness (*Extract*), 295.
Seifert and Müller, Drs.—Manual of clinical diagnosis (*Review*), 56.
Sinclair, Dr. W. J.—Gonorrhœal infection in women (*Review*), 135.
Skin, Diseases of—Dr. H. R. Crocker (*Review*), 201; Dr. W. A. Jamieson (*Review*), 138.
Smith, Dr. R. S.—Phthisis and the germ theory, 225; two cases of phthisis, 185.
Spinal cases—Dr. J. M. Clarke, 120.
Spinal paralysis (*Extract*), 295.
Squire, Dr. W.—Medical essays (*Review*), 288.
Stephens, L.—Case of suicidal perforation of stomach, 113.
Stewart, W. R. H.—Diseases of ear (*Review*), 284.
Stomach, Case of suicidal perforation of—L. Stephens, 113.
Sulphonal (*Extract*), 293.
Surgery, A treatise on—T. Holmes (*Review*), 276.
Surgical operations, Manual of—A. E. J. Barker (*Review*), 53.
Swanzy, Dr. H. R.—Diseases of eye (*Review*), 200.
Swayne, Dr. J. G.—The hour of delivery, 174.

Syphilis, Cases of constitutional—W. J. Penny, 45.

Testicle, Death from blow on (*Extract*), 69.
Therapeutic notes, Pocket — Ferris & Co. (*Review*), 139.
Thin, Dr. G.—Ringworm (*Review*), 279.
Thompson, Sir H. — Diseases of urinary organs, (*Review*), 289.
Thyroid body, Surgery of—W. H. Harsant, 265.
Tongue, Affections of—W. J. Penny, 37.
Tongue, Varix of (*Extract*), 148.
Tonsils, Tuberculosis of (*Extract*), 149.
Tubercle-bacillus (*Extract*), 294.
Tuberculosis in infants (*Extract*), 63.
Tumours, Intracranial — Dr. B. Bramwell (*Review*), 283.
Tympanites—Dr. J. W. Ogle (*Review*), 283.

Urachus, Case of patent—W. J. Penny, 36.
Urethral discharges (*Extract*), 69.
Urinary organs, Diseases of — Sir H. Thompson (*Review*), 289.
Uterus, Cancer of—Dr. J. Williams (*Review*), 134.

Vocal-cords, Nodules on (*Extract*), 70.

Waldo, Dr. H.—Case of Raynaud's disease, 272.
Williams, Dr. J.—Cancer of uterus (*Review*), 134.
Williams, Dr. P. W.—Case of morbus cæruleus, 183.
Williams, W. R.—Cancer and tumour formation (*Review*), 203.

Year-book of treatment (*Review*), 55.

---

J. W. Arrowsmith, Printer, Quay Street, Bristol.

### Bristol Medico-Chirurgical Society.

---

### PAST PRESIDENTS.

1874—75. FREDERICK BRITTAN, M.D., B.A. Dub.

1875—76. ROBERT WILLIAM COE.

1876—77. SAMUEL MARTYN, M.D. Ed.

1877—78. AUGUSTIN PRICHARD, M.D. Berlin.

1878—79. HENRY EDWARD FRIPP, M.D. Würzburg.

1879—80. WILLIAM MICHELL CLARKE.

1880—81. JOSEPH GRIFFITHS SWAYNE, M.D. Lond.

1881—82. EDWARD LONG FOX, M.D. Oxon.

1882—83. JAMES GEORGE DAVEY, M.D. St. And.

1883—84. WILLIAM JOHNSTONE FYFFE, M.D. Dub.

1884—85. GEORGE FORSTER BURDER, M.D. Aberd.

1885—86. WILLIAM HENRY SPENCER, M.D., M.A. Cantab.

1886—87. FRANCIS POOLE LANSDOWN.

1888.

### LIST OF SUBSCRIBERS

TO THE

### Bristol Medico-Chirurgical Journal.

*Those marked * are Members of the Society.*

| *Name.* | *Residence.* |
| --- | --- |
| ABERCROMBIE, JOHN, M.D. Cantab.... ... ... | 23 Upper Wimpole Street, London, W. |
| ACKLAND, J. MACKNO ... ... ... ... ... ... | 24 Southernhay, Exeter. |
| ACKLAND, W. H., M.D. St. And. ... ... ... | Bideford. |
| *ACKLAND, W. R. ... ... ... ... ... ... ... | 5 Rodney Place, Clifton. |
| ADAMS, GEORGE ... ... ... ... ... ... ... | 11 Westbourne Place, Clifton. |
| *ADAMS, JOHN BARFIELD... ... ... ... ... ... | 3 Selwood Villas, Bristol. |
| ALEXANDER, JAMES, M.D., M.Ch., B.A. Dub. | Bishop's Place, Paignton, Devonshire. |
| *ALFORD, GEORGE ERNEST ... ... ... ... ... | 5 Royal Crescent, Weston-super-Mare. |
| ALFORD, HENRY J., M.D. Lond. ... ... ... | 2 Marlborough Terrace, Taunton. |
| ANDREWS, ONSLOW, M.D. St. And.... ... ... | 159 White Ladies Road, Bristol. |
| ANSTIE, THOMAS B. ... ... ... ... ... ... | 21 Northgate Street, Devizes. |
| *ATCHLEY GEORGE F., M.B. Lond.... ... ... | Tyndall's Park, Bristol. |
| BAKER, A. de W. ... ... ... ... ... ... ... | 2 Lawn Terrace, Dawlish. |
| *BARCLAY, W. M. ... ... ... ... ... ... ... | General Hospital, Bristol. |
| BARETTI, T. G. L'ENARDI ... ... ... ... ... | 49 Royal York Crescent, Clifton. |
| *BARON, BARCLAY J., M.B., C.M. Ed. ... ... | 16 White Ladies Road, Clifton. |
| BASSET, WALTER ... ... ... ... ... ... ... | The Infirmary, Newport, Mon. |
| BATTEN, RAYNER W., M.D. Lond. ... ... ... | 1 Brunswick Square, Gloucester. |
| BATTEN, W. SMITH ... ... ... ... ... ... | Curzon House, Calne. |
| BEALE, LIONEL S., M.B. Lond., F.R.S. ... | 61 Grosvenor Street, London, W. |
| BEATSON, W. B., M.D. St. And. ... ... ... | 11 Cavendish Place, Bath. |
| *BEDDOE, JOHN, M.D. Ed., B.A. Lond., F.R.S. | The Manor House, Clifton. |
| BENHAM, HARRY A., M.D., C.M. Aberd. ... | Asylum, Stapleton, Gloucestershire. |
| BENNETT, W. S. ... ... ... ... ... ... ... | Escot, Penzance. |
| BERRY, F. C., M.D., B.Ch., B.A. Dub. ... ... | Lyn Cottage, Lynton. |
| BISDÉE, ALFRED J.... ... ... ... ... ... ... | Eversleigh, Banwell, Somersetshire. |
| *BLACKER, A. E. ... ... ... ... ... ... ... | 5 Unity Street, Bristol. |
| BLACKER, E. ... ... ... ... ... ... ... ... | Midsomer Norton. |
| *BLAGG, ARTHUR F. ... ... ... ... ... ... | 6 West Mall, Clifton. |
| *BOARD, EDMUND C. ... ... ... ... ... ... | 7 Caledonia Place, Clifton. |
| BOWER, ERNEST D. ... ... ... ... ... ... | 1 Norfolk Terrace, Gloucester. |
| BOYLE, THOMAS ... ... ... ... ... ... ... | New Quay, Cornwall. |
| BOYS, ARTHUR HENRY ... ... ... ... ... ... | Chequer Street, St. Alban's. |
| BRAMWELL, J. W., M.D. Ed. ... ... ... ... | 6 Bayshill Villas, Cheltenham. |
| BRIGHT, J. A. ... ... ... ... ... ... ... ... | Glastonbury. |

### LIST OF SUBSCRIBERS.

| | |
|---|---|
| Broom, John, M.D., Ch.D., Obst D. Brussels | St. Paul's Road, Clifton. |
| Brown, G. Arthur ... ... ... ... ... ... ... | Tredegar. |
| *Brown, William, M.B., C.M. Glasg. ... ... | Fishponds, Gloucestershire. |
| Browne, A. E. Newbury ... ... ... ... ... ... | Burbage, Wiltshire. |
| Browne, G. Henry ... ... ... ... ... ... ... | The Hermitage, Brynmawr, Breconshire. |
| Browne, J. Walton, M.D., B.A. Qu. Univ. I. | 10 College Square North, Belfast. |
| Browne, Lennox ... ... ... ... ... ... ... ... | 36 Weymouth Street, London, W. |
| Bubb, John ... ... ... ... ... ... ... ... ... | 6 Royal Crescent, Cheltenham. |
| *Burder, George F., M.D. Aberd. ... ... ... | 63 Oakfield Road, Clifton. |
| Burges, R. E., M.D., M.Ch., B.A. Qu. Univ. I. | Kettering. |
| Burroughs, E. F. H. ... ... ... ... ... ... ... | 64 Bristol Road, Weston-super-Mare. |
| *Bush, J. Paul ... ... ... ... ... ... ... ... ... | 13 Lansdown Place, Clifton. |
| *Calder, F. ... ... ... ... ... ... ... ... ... ... | Medical School, Bristol. |
| Cann, Francis M. ... ... ... ... ... ... ... ... | Sefton House, Dawlish. |
| Carey, J., M.D. Lond. ... ... ... ... ... ... ... | 2 Hamdon Villas, Taunton. |
| Carlyon, Thomas B. ... ... ... ... ... ... ... | Redruth. |
| *Carr, Alexander ... ... ... ... ... ... ... ... | Wells Road, Bristol. |
| Carter, R. W., M.D. Calcutta ... ... ... ... | 1 Bank Buildings, Weymouth. |
| *Carter, W. F. ... ... ... ... ... ... ... ... ... | 48 Coronation Road, Bristol. |
| Cave, Edward J., M.D. Lond. ... ... ... ... | Royal Infirmary, Newcastle-on-Tyne. |
| Clark, Thomas ... ... ... ... ... ... ... ... | Blair Lodge, Minehead, Somersetshire. |
| Clarke, Arthur, B.A. Lond. ... ... ... ... ... | Street, Somersetshire. |
| Clarke, Fred. H. ... ... ... ... ... ... ... ... | Holly Lodge, Chillington, Kingsbridge. |
| *Clarke, John Michell, M.B., M.A. Cantab. | 2 York Buildings, Clifton. |
| Clay, Challoner ... ... ... ... ... ... ... ... | Manor House, Fovant. |
| Clay, R. Hogarth, M.D. Ed. ... ... ... ... ... | 4 Windsor Villas, Plymouth. |
| Coates, Harcourt ... ... ... ... ... ... ... | 17 Endless Street, Salisbury. |
| Coathupe, Edwin W. ... ... ... ... ... ... ... | 7 Clifton Park, Clifton. |
| *Coe, R. W. ... ... ... ... ... ... ... ... ... | 14 Berkeley Square, Bristol. |
| Coker, T. V. ... ... ... ... ... ... ... ... ... | 2 Chesterfield Place, Clifton. |
| Cole, Thomas, M.D. Lond. ... ... ... ... | 18 Circus, Bath. |
| *Collins, H. W. ... ... ... ... ... ... ... ... | Wrington, Somersetshire. |
| Collyns, Robert J. ... ... ... ... ... ... ... | Dulverton, Somersetshire. |
| Colman, Thomas J., M.D. Ed. ... ... ... ... | Elmgrove Road, Bristol. |
| Colmer, Ptolemy S. H., M.D. Dur. ... ... | South Street House, Yeovil. |
| Cooke, A. S. ... ... ... ... ... ... ... ... ... | Badbrook House, Stroud. |
| Cooper, Herbert ... ... ... ... ... ... ... ... | Hampstead, London, N.W. |
| *Corbould, George G. ... ... ... ... ... ... | 22 Berkeley Square, Bristol. |
| *Cotton, William, M.B., C.M. Ed. ... ... | 227 Gloucester Road, Bristol. |
| Cowan, F. S. ... ... ... ... ... ... ... ... ... | 27 Queen Square, Bath. |
| Cox, William I. ... ... ... ... ... ... ... ... | Hawkesbury-Upton. |
| Crespi, Alfred J. H. ... ... ... ... ... ... | Poole Road, Wimborne. |
| *Crisp, Nathaniel ... ... ... ... ... ... ... ... | Keynsham, Somersetshire. |
| *Cross, F. Richardson, M.B. Lond. ... ... | Chandos Villa, Clifton. |
| *Crossman, Edward, M.D. Dur. ... ... ... | Hambrook, Gloucestershire. |
| Currie, Andrew S., M.D. Ed. ... ... ... ... | The Moorlands, Lydney. |
| *Dacre, John ... ... ... ... ... ... ... ... ... | 1 Leicester Place, Clifton. |
| *Daly, George H., M.D. St. And. ... ... ... | Burley House, Chippenham. |
| Daniel, T. P. ... ... ... ... ... ... ... ... ... | Beaminster, Dorsetshire. |
| *Davey, James G., M.D. St. And. ... ... ... | Ellesmere House, Shepperton. |
| *Davies, D. S., M.B. Lond. ... ... ... ... | 60 Oakfield Road, Clifton. |
| Davies, E. Cluneglas ... ... ... ... ... ... | Pontfaen, Lampeter, Cardiganshire. |
| Davies, John ... ... ... ... ... ... ... ... ... | 38 Gay Street, Bath. |

### LIST OF SUBSCRIBERS.

| | |
|---|---|
| Davies, John W. | Ebbw Vale, Monmouthshire. |
| Davis, Robert | 14 St. James's Square, Bath. |
| Davis, Theodore, M.D. Lond. | Beachcroft, Clevedon. |
| Day, Percy Howard | Stalmine Lodge, Poulton-le-Fylde |
| Deas, P. Maury, M.B., M.S. Lond. | Wonford House, Exeter. |
| Dening, Edwin | Stow-on-the-Wold. |
| Devonald, Alfred | Gellingham House, Carmarthen. |
| *Dew, Henry R. | 25 Berkeley Square, Bristol. |
| *Dobson, Nelson C. | 27 Victoria Square, Clifton. |
| *Dowson, C. H. | Tyndall's Park, Bristol. |
| *Dowson, W., M.B., B.C., M.A. Cantab. | Great George Street, Bristol. |
| Doyne, Robert W. | Southbourne, Oxford. |
| Dunbar, Eliza L. Walker, M.D. Zürich | 9 Oakfield Road, Clifton. |
| *Duncan, J. G. | Foresters' Dispensary, Bristol. |
| Duncan, William | Ridgeway, Frome. |
| | |
| Eadon, W. F. Bailey | Hambrook, Gloucestershire. |
| *Eager, Reginald, M.D. Lond. | Northwoods, Winterbourne. |
| East, Edward | 16 Upper Berkeley Street, London, W. |
| Eddowes, Charles | Maddington, Devizes. |
| Edgeworth, T. F. | 1 Stokes Croft, Bristol. |
| Edwardes, Thomas | Llansaintffraid, Montgomeryshire. |
| *Elliott, Christopher, M.D., B.A. Dub. | 88 Pembroke Road, Clifton. |
| Ellis, Henry V., M.B., C.M. Aberd. | Ty Bryn, Reynoldstone. |
| Embleton, Dennis C. | St. Wilfrid's, Bournemouth. |
| *Etches, W. R. | 13 Dowry Square, Clifton. |
| Evans, G. M. | Bridport. |
| Evans, J. Fenton, M.B., C.M. Ed. | 6 Whitehall Yard, London, S.W. |
| Evans, W. G. | Beckington. |
| *Ewens, John | 14 White Ladies Road, Clifton. |
| | |
| Fairbanks, W., M.D., C.M. Ed. | Wells, Somersetshire. |
| Fawcett, W. J., M.B., M.Ch., B.A. Dub. | Shirehampton. |
| *Fendick, R. George | 3 Claremont Place, Clifton. |
| Fernie, James | Stratton St. Margaret. |
| Finch, Thomas, M.D. St. And. | Westville, St. Mary Church. |
| Fisher, Fred. B. | West Walk, Dorchester. |
| Fletcher, Henry Studd | Lydbrook. |
| Ford, Joseph | Wedmore. |
| Forty, D. H. | Wotton-under-Edge. |
| Fothergill, J. Milner, M.D. Ed. | 3 Henrietta Street, London, W. |
| Fox, Arthur E. W., M.B., C.M. Ed. | 16 Gay Street, Bath. |
| *Fox, Bonville B., M.D., M.A. Oxon. | Brislington. |
| Fox, Charles Henry, M.D. St. And. | The Beeches, Brislington. |
| *Fox, E. Long, M.D. Oxon. | Church House, Clifton. |
| Fraser, D. A., M.D. Brussels | Pomeroy House, Totnes. |
| Fraser, Græme B. | Belvidere, Weston-super-Mare. |
| Freeman, Henry W. | 24 Circus, Bath. |
| Fry, John B. | Swindon, Wiltshire. |
| Furse, Edwin | South Molton. |
| *Fyffe, W. Johnstone, M.D., B.A. Dub. | 2 Rodney Place, Clifton. |
| | |
| Gabb, J. Edward | London Road, Cheltenham. |
| Gamble, C. H. | Barnstaple. |
| Gardiner, George | 1 Redcliff Hill, Bristol. |

### LIST OF SUBSCRIBERS.

| | |
|---|---|
| *GIBBS, ALFRED N. GODBY ... ... ... ... ... | 27 Chandos Road, Bristol. |
| GIBBS, ROBERT ... ... ... ... ... ... ... ... | Orchard House, Teignmouth. |
| GILBERTSON, RICHARD ... ... ... ... ... ... | Aberystwith. |
| GILL, JOHN, M.D. Brussels ... ... ... ... ... | 31 Apsley Road, Clifton. |
| *GLOAG, G. A. ... ... ... ... ... ... ... ... ... | 7 College Green, Bristol. |
| GOODDEN, WYNDHAM C. ... ... ... ... ... | 86 Stapleton Road, Bristol. |
| GOODING, J. CALLENDER, M.D. Ed. ... ... | Berkeley Street, Cheltenham. |
| GOODRIDGE, HENRY F. A., M.D. Lond. ... | 10 Brock Street, Bath. |
| GOSS, T. BIDDULPH ... ... ... ... ... ... | 1 Circus, Bath. |
| GOULD, A. PEARCE, M.B., M.S. Lond. ... ... | 16 Queen Anne Street, London, W. |
| GRACE, ALFRED ... ... ... ... ... ... ... | Chipping Sodbury. |
| GRACE, EDWARD MILLS ... ... ... ... ... | Thornbury, Gloucestershire. |
| GRACE, HENRY ... ... ... ... ... ... ... ... | Kingswood, Bristol. |
| GRACE, W. G. ... ... ... ... ... ... ... ... ... | 57 Stapleton Road, Bristol. |
| GRAY, F. A. ... ... ... ... ... ... ... ... ... | Ottery St. Mary. |
| GREEN, F. K. ... ... ... ... ... ... ... ... ... | 3 Gay Street, Bath. |
| GREGORY, GEORGE S. ... ... ... ... ... ... | Southampton Row, London. |
| *GRIFFITHS, L. M. ... ... ... ... ... ... ... | 9 Gordon Road, Clifton. |
| GRIGOR, MACADAM ... ... ... ... ... ... ... | Alexandra Avenue, London, S.W. |
| GROTE, G. W., M.B. Toronto ... ... ... ... | 24 St. Andrew's Crescent, Cardiff. |
| GUINNESS, T. A. ... ... ... ... ... ... ... ... | Bampton, Devonshire. |
| | |
| *HAILES, CLEMENTS D. G., M.D., C.M. Ed. ... | 11 King's Parade, Clifton. |
| HALE, EDMUND T. ... ... ... ... ... ... ... ... | The Grange, Chew Magna. |
| HARPER, JOSEPH ... ... ... ... ... ... ... ... | Bear Street, Barnstaple. |
| *HARRISON, A. J., M.B. Lond. ... ... ... ... | Guthrie Road, Clifton. |
| *HARSANT, W. H. ... ... ... ... ... ... ... ... | 16 Pembroke Road, Clifton. |
| HAWKINS, CÆSAR F. ... ... ... ... ... ... | 7 Berkeley Square, Bristol. |
| HAYDON, EDGAR, M.B., C.M. Glasg. ... ... | The Laurels, Newton Abbot. |
| HAYMAN, A. G.... ... ... ... ... ... ... ... ... | 1 Pembroke Road, Clifton. |
| HEANE, W. C. ... ... ... ... ... ... ... ... ... | Cinderford. |
| HEARDER, G. J., M.D. St. And.... ... ... ... | Joint Counties Asylum, Carmarthen. |
| *HEAVEN, JOHN C. ... ... ... ... ... ... ... ... | 2 Queen Square, Bristol. |
| *HERAPATH, C. K. C. ... ... ... ... ... ... ... | 11 Brunswick Square, Bristol. |
| *HETLING, H. E. ... ... ... ... ... ... ... ... | Stapleton Road, Bristol. |
| HETT, GEOFFREY, M.D. Ed. ... ... ... ... | 100 Inverness Terrace, London, W. |
| HICHENS, JAMES STACEY ... ... ... ... ... | 17 Green Lane, Redruth. |
| HILLS, FREDERIC F. ... ... ... ... ... ... ... | 199 Cheltenham Road, Bristol. |
| HINTON, JOSEPH ... ... ... ... ... ... ... ... | The Chestnuts, Warminster. |
| HOBLYN, FRANCIS PARKER ... ... ... ... ... | 10 Bladud Buildings, Bath. |
| HOOKER, CHARLES P. ... ... ... ... ... ... ... | 104 Dyer Street, Cirencester. |
| HOPKINS, H. CULLIFORD ... ... ... ... ... | 4 Gay Street, Bath. |
| HOSPITAL, BRISTOL GENERAL (*Sec.*, W. THWAITES). | |
| HOWELLS, WILLIAM, M.B., C.M. Glasg. ... | Church House, Talgarth, Breconshire |
| HOWSE, WILLIAM ... ... ... ... ... ... ... ... | 8 London Street, Swindon, Wiltshire. |
| HOWSIN, E. ARTHUR, M.D. St. And. ... ... | Stroud. |
| HUDSON, H. R. ... ... ... ... ... ... ... ... | 182 Commercial Road, Newport, Mon. |
| HUNT, A. D. ... ... ... ... ... ... ... ... ... | Millbrook House, Chagford. |
| HYATT, BROWNLOW N. ... ... ... ... ... ... | Park View, Shepton Mallet. |
| | |
| *IMLAY, G. A., M.B., C.M. Aberd. ... ... ... | 4 Apsley Villas, Bristol. |
| INFIRMARY, BRISTOL ROYAL (*Librarian*, Dr. JAMES SWAIN). | |
| INSTITUTION, LIVERPOOL MEDICAL (*Hon. Treas.*, Dr. JAMES BARR). | |
| ISAAC, G. WASHINGTON, M.B., C.M. Ed. ... | 7 Mornington Crescent, London, N.W. |

| | |
|---|---|
| Jackson, J. B., M.D., M.Ch. Roy. Univ. I. ... | 56 Lemon Street, Truro. |
| James, J. Bowen ... ... ... ... ... ... ... | Woodlands, Aberaman, Aberdare. |
| Jessop, Charles Moore ... ... ... ... ... | 98 Sutherland Avenue, London, W. |
| Jones, E. R. ... ... ... ... ... ... ... ... | Cilgynlle-fawr, New Quay, Cardiganshire. |
| Jones, Evan ... ... ... ... ... ... ... ... | Ty-mawr, Aberdare. |
| Jones, W. R., M.D., C.M. Glasg. ... ... ... | Senny Bridge, Breconshire. |
| Joynes, Francis James ... ... ... ... ... | Eagle House, Dursley. |
| *Keall, W. P. ... ... ... ... ... ... ... ... | Coronation Road, Bristol. |
| Kelly, C. I. ... ... ... ... ... ... ... ... | St. Martin's, Eyrecourt, Galway. |
| Kempe, Arthur W. ... ... ... ... ... ... | 9 Southernhay, Exeter. |
| Kendall, Bernard ... ... ... ... ... ... | Elm Grove, Tenby. |
| Kerr, Elias W., M.B., M.Ch., B.A. Dub. ... | Cerne Abbas. |
| Kesteven, W. B., M.D. St. And. ... ... ... | The Cedars, Cheshunt. |
| King, Louis J.... ... ... ... ... ... ... ... | 10 Russel Street, Bath. |
| Kinneir, R. ... ... ... ... ... ... ... ... | The Tower House, Malmesbury. |
| Kirkman, J. Miller ... ... ... ... ... ... | Wootton Bassett. |
| Laing, W. A. Gordon, M.D., C.M. Glasg. ... | Boutport Street, Barnstaple. |
| *Lansdown, F. Poole ... ... ... ... ... ... | 19 White Ladies Road, Clifton. |
| *Lawrence, A. E. Aust, M.D., C.M. Aberd. . | 19 Richmond Hill, Clifton. |
| *Lawrence, Henry ... ... ... ... ... ... | 9 Park Row, Bristol. |
| Leach, J. Comyns, M.D. Dur., B.Sc. Lond. . | Sturminster Newton. |
| Leamon, Michael T. ... ... ... ... ... ... | 1 Bedford Villas, Tavistock. |
| *Lees, E. Leonard, M.B., C.M. Ed. ... ... | Redland Road, Bristol. |
| Lewellyn, John ... ... ... ... ... ... ... | Caerphilly. |
| Library, Bristol Museum and ... ... ... | Queen's Road, Bristol. |
| Library, Cheltenham ... ... ... ... ... | 5 Royal Crescent, Cheltenham. |
| *Lidderdale, James ... ... ... ... ... ... | Portishead. |
| Liddon, William, M.B. Lond.... ... ... ... | Church Square, Taunton. |
| Lilley, G. Herbert, M.D. Brussels ... ... | H.M. Convict Prison, Portland. |
| Lister, Sir Joseph, Bart., F.R.S. ... ... ... | 12 Park Crescent, London, N.W. |
| Lloyd, Walter E. ... ... ... ... ... ... | 60 York Road, Bristol. |
| Lodge, John ... ... ... ... ... ... ... ... | Keynsham, Somersetshire. |
| *Logan, Frederic T. B. ... ... ... ... ... | Dean Lane, Bristol. |
| Lovell, W. Day ... ... ... ... ... ... ... | The Abbey, Bradford-on-Avon. |
| Lowe, T. Pagan ... ... ... ... ... ... ... | 19 Alfred Street, Bath. |
| Luckham, L. S. ... ... ... ... ... ... ... | General Infirmary, Salisbury. |
| *Luffman, W. C. ... ... ... ... ... ... ... | 8 Cotham New Road, Bristol. |
| *Lyons, A. de Courcy, M.B., C.M. Aberd. ... | Henley Lodge, Yatton, Somersetshire. |
| MacDonald, P. W., M.D., C.M. Aberd. ... | County Asylum, Dorchester. |
| Maclean, J. Campbell, M.B., C.M. Ed. ... | Swindon House, Swindon, Wiltshire. |
| Maclean, Kenneth ... ... ... ... ... ... | Greek Street, Stockport. |
| Manning, G. H., M.B., B.Ch., B.A. Dub. ... | Combe Martin. |
| Manning, H. J., B.A. Lond. ... ... ... ... | Laverstock House, Salisbury. |
| Marley, Henry F. ... ... ... ... ... ... | The Nook, Padstow, Cornwall. |
| Marsh, Charles J. ... ... ... ... ... ... | Yeovil. |
| Marsh, O. E. Bulwer ... ... ... ... ... | Ventnor House, Newport, Mon. |
| *Marshall, Henry, M.D. Ed., F.R.S.E. ... | 28 Caledonia Place, Clifton. |
| *Martin, E. F., M.B. Ed. ... ... ... ... ... | Victoria House, Weston-super-Mare. |
| Martin, Theodore ... ... ... ... ... ... | Temple Cloud. |
| Mason, Frederick ... ... ... ... ... ... | 20 Belmont, Bath. |

| | |
|---|---|
| MASON, S. B. ... ... ... ... ... ... ... ... ... | 12 Park Terrace, Pontypool. |
| MASON, WILLIAM ... ... ... ... ... ... ... ... | Sedgemoor Cottage, St. Austell. |
| *MATTHEWS, CHARLES E. ... ... ... ... ... ... | 31 Pembroke Road, Clifton. |
| *MAYOR, T. O. ... ... ... ... ... ... ... ... ... | 40 Apsley Road, Clifton. |
| McNICOLL, JOHN ... ... ... ... ... ... ... ... | High Street, Poole. |
| MEREDITH, JOHN, M.D. Ed. ... ... ... ... | Wellington, Somersetshire. |
| METFORD, J. SEYMOUR ... ... ... ... ... | 31 Berkeley Square, Bristol. |
| MICHELL, GEORGE A. ... ... ... ... ... ... ... | Redruth. |
| MILNE, JOHN, M.B., C.M. Aberd. ... ... ... | 97 Clarence Road, Bristol. |
| MILWARD, JAMES ... ... ... ... ... ... ... ... | 54 Charles Street, Cardiff. |
| MORGAN, FREDERICK ... ... ... ... ... ... | Uffculme. |
| MORGAN, JOHN ... ... ... ... ... ... ... ... ... | Langport, Somersetshire. |
| MORGAN, WILLIAM, M.D. Aberd. ... ... ... | 16 Edwards Terrace, Cardiff. |
| MORRIS, J. A. ... ... ... ... ... ... ... ... ... | Caerleon, Monmouthshire. |
| MOYNAN, W. ARTHUR, M.D., M.Ch. Qu. Univ. I. | St. Ann's Heath, Chertsey. |
| MUNDEN, CHARLES ... ... ... ... ... ... ... ... | Ilminster. |
| MURRAY, WILLIAM, M.B., C.M. Ed. ... ... | Staple Hill, Gloucestershire. |
| NAPIER, T. W. A., M.B., C.M. Aberd. ... ... | Church Street, Egremont, Cheshire. |
| *NEWMAN, CHARLES ... ... ... ... ... ... ... ... | 19 Portland Square, Bristol. |
| *NEWNHAM, W. H. C., M.B., M.A. Cantab. ... | General Hospital, Bristol. |
| NEWSTEAD, JAMES ... ... ... ... ... ... ... ... | 9 York Place, Clifton. |
| NEWTON, CHARLES JOHN ... ... ... ... ... ... | Oriel Lodge, Cheltenham. |
| NICHOLSON, T. D., M.D., C.M. Ed. ... ... | 2 White Ladies Road, Clifton. |
| NORMAN, GEORGE ... ... ... ... ... ... ... ... | 12 Brock Street, Bath. |
| NORMAN, J. H. ... ... ... ... ... ... ... ... ... | Goodleigh, Winkleigh, Devonshire. |
| NORMAN, J. W. ... ... ... ... ... ... ... ... ... | Edde Cross House, Ross. |
| *NORTON, J. A., M.D., C.M. Aberd. ... ... ... | 8 Redcliff Hill, Bristol. |
| *NORTON, T. CHALMERS ... ... ... ... ... | 12 King Square, Bristol. |
| NOURSE, W. J. CHICHELE ... ... ... ... ... | Morchard Bishop, Devonshire. |
| OGSTON, PROF. ALEXANDER, M.D., C.M. Aberd. | 252 Union Street, Aberdeen. |
| OLLERHEAD, T. J. ... ... ... ... ... ... ... ... | Hillbury, Minehead, Somersetshire. |
| *ORMEROD, HENRY ... ... ... ... ... ... ... | Westbury-on-Trym. |
| PADLEY, GEORGE ... ... ... ... ... ... ... ... | 2 Northampton Terrace, Swansea. |
| PAGE, G. SHEPLEY ... ... ... ... ... ... ... ... | 78 Old Market Street, Bristol. |
| PAINE, WILLIAM HENRY, M.D. Aberd. ... ... | Stroud. |
| PALM, THEOBALD A., M.D., C.M., M.A. Ed. . | Greenhill, Thorncombe. |
| PALMER, F. CRADDOCK ... ... ... ... ... ... | The Corderries, Chalford. |
| *PARKER, GEORGE, M.D., M.A. Cantab. ... ... | Dispensary, Bristol. |
| PARRY, J. H. ... ... ... ... ... ... ... ... ... | 99 Ashley Road, Bristol. |
| PARSLOE, H. H. ... ... ... ... ... ... ... ... | Black Torrington, Devonshire. |
| *PARSON, T. COOKE ... ... ... ... ... ... ... | 21 White Ladies Road, Bristol. |
| PARSONS, FRANCIS J. C. ... ... ... ... ... | King Square, Bridgwater. |
| PARSONS, FREDERICK ... ... ... ... ... ... | Common Hill, Frome. |
| PAULI, G. C. P. ... ... ... ... ... ... ... ... | 240 York Road, Bristol. |
| PAULL, JOSIAH ... ... ... ... ... ... ... ... ... | Camborne, |
| PENNY, F. ... ... ... ... ... ... ... ... ... ... | The Infirmary, Doncaster. |
| *PENNY, W. J. ... ... ... ... ... ... ... ... ... | 42 Caledonia Place, Clifton. |
| PENTLAND, YOUNG J. ... ... ... ... ... ... | 11 Teviot Place, Edinburgh. |
| PERKINS, J. H., M.D. New York ... ... ... | 14 Victoria Square, Clifton. |

| | |
|---|---|
| Peskett, A. W. C., M.B., B.C., M.A. Cantab. | Burnham, Somersetshire. |
| Phelps, W. Harford G., M.D. Aberd. ... | Beachfield, Weston-super-Mare. |
| Phillips, E. England ... ... ... ... ... ... | Broadwater House, Southend. |
| Philpots, Edward P., M.D., C.M. Aberd. ... | Bourne Hall, Bournemouth. |
| Philpots, John R. ... ... ... ... ... ... | Moorcroft, Parkstone, Dorsetshire. |
| *Pickering, C. F. ... ... ... ... ... ... ... | 12 Berkeley Square, Bristol. |
| Pippette, Walter... ... ... ... ... ... ... | Wilton, Wiltshire. |
| Pizey, G. F. P.... ... ... ... ... ... ... ... | Rydal Villa, Clevedon. |
| Plaister, W. H. ... ... ... ... ... ... ... | High Road, Tottenham. |
| *Pountney, W. E., M.D., C.M. Ed. ... ... | 107 White Ladies Road, Bristol. |
| Powell, William, M.B. Lond. ... ... ... | Hill Garden, Torquay. |
| Price, William, M.B. Lond. ... ... ... ... | 40 Park Place, Cardiff. |
| *Prichard, Arthur W.... ... ... ... ... ... | Gordon Road, Clifton. |
| *Prichard, Augustin, M.D. Berlin ... ... ... | 4 Chesterfield Place, Clifton. |
| *Prichard, J. E., M.B., B.A. Oxon. ... ... | 243 Cheltenham Road, Bristol. |
| Prideaux, T. Engledue P. ... ... ... ... | Wellington, Somersetshire. |
| *Pring, Arthur, B.A. Cantab. ... ... ... ... | 4 Pembroke Road, Clifton. |
| Prowse, Albert P. ... ... ... ... ... ... | Yennadon, Horrabridge, Devonshire. |
| *Prowse, Arthur B., M.D. Lond. ... ... ... | 5 Lansdown Place, Clifton. |
| Pullin, Bingley G. ... ... ... ... ... ... | Sidmouth. |
| | |
| *Rahilly, J. R.... ... ... ... ... ... ... ... | Horfield Barracks, Bristol. |
| Randolph, Charles ... ... ... ... ... ... | St. Michael's, Milverton, Somersetshire. |
| Rawlings, John Adams ... ... ... ... ... | 4 Northampton Terrace, Swansea. |
| Redwood, T. Hall, M.D., C.M., M.A. Dur.... | The Lawn, Rhymney, Monmouthshire. |
| Rendall, John... ... ... ... ... ... ... ... | Forestside, Lymington. |
| Riddell, Andrew W. ... ... ... ... ... ... | Westbury-on-Trym. |
| Robathan, G. B. ... ... ... ... ... ... ... | The Grove, Risca. |
| Rogers, George, M.D. St. And. ... ... ... | 17 Portland Square, Bristol. |
| Rolston, G. T. ... ... ... ... ... ... ... | 8 Osborne Villas, Stoke, Devonport. |
| Rolston, John R. ... ... ... ... ... ... ... | 5 St. Aubyn Street, Devonport. |
| *Ross, R. A. ... ... ... ... ... ... ... ... | Lodway Villa, Pill. |
| Rossiter, George F., M.B. Lond. ... ... | Cairo Lodge, Weston-super-Mare. |
| *Roué, W. Barrett, M.D., M.S. Dur. ... ... | 2 Buckingham Place, Clifton. |
| Row, Charles ... ... ... ... ... ... ... | Lostwithiel. |
| Row, William ... ... ... ... ... ... ... ... | Lerrin, Lostwithiel. |
| Rowe, Edmund L.... ... ... ... ... ... ... | County Asylum, Gloucester. |
| *Roxburgh, Robert, M.B. Ed. ... ... ... ... | 1 Victoria Buildings, Weston-super-Mare. |
| Royle, John Frederick S., M.B., C.M. Aberd. | High Street, Milborne Port. |
| *Rudge, C. King ... ... ... ... ... ... ... | 145 White Ladies Road, Bristol. |
| *Rudge, H. T. ... ... ... ... ... ... ... ... | 5 Colston Parade, Bristol. |
| Rundle, Edmund ... ... ... ... ... ... ... | Royal Cornwall Infirmary, Truro. |
| Rundle, Henry ... ... ... ... ... ... ... | 11 Clarence Parade, Southsea, Hampshire. |
| Runnalls, H. Boyle ... ... ... ... ... ... | Laurel Bank, Saltash, Cornwall. |
| Russell, M. W. H. ... ... ... ... ... ... | 6 Whitehall Yard, London, S.W. |
| | |
| Scott, James, M.B., C.M. Ed.... ... ... ... | 35 Wolverhampton Road, Stafford. |
| Scott, Richard J. H. ... ... ... ... ... ... | 13 Bladud Buildings, Bath. |
| Scott, W. G., M.B., C.M. Ed. ... ... ... | Newton Abbot. |
| Searle, George C. ... ... ... ... ... ... | Brixham, Devonshire. |
| Searle, Richard Burford ... ... ... ... | St. Just. |
| *Semple, H. F.... ... ... ... ... ... ... ... | Ladismith, Cape Town. |
| Seward, W. J., M.B. Lond. ... ... ... ... | Asylum, Colney Hatch, London, N. |
| Sewart, J. H. ... ... ... ... ... ... ... ... | Lostwithiel. |

### LIST OF SUBSCRIBERS.

| | |
|---|---|
| Shapter, Lewis, M.D., B.A. Cantab. ... ... | The Barnfield, Exeter. |
| *Shaw, J. E., M.B., C.M. Ed. ... ... ... ... | 11 Lansdown Place, Clifton. |
| Sheen, A., M.D. St. And. ... ... ... ... ... | 23 Newport Road, Cardiff. |
| Sheldon, T. Steele, M.B. Lond.... ... ... | Parkside House, Macclesfield. |
| *Sheppard, E. J. ... ... ... ... ... ... ... | 16 Park Place, Clifton. |
| *Shorland, Walter E. ... ... ... ... ... | 189 Cheltenham Road, Bristol. |
| Shortridge, Thomas Wood, M.D. Brussels | Honiton. |
| Sincock, J. Bain ... ... ... ... ... ... ... | Friarn Street, Bridgwater. |
| Skeate, Edwin ... ... ... ... ... ... ... | 16 Paragon, Bath. |
| *Skelton, Henry, M.D. St. And. ... ... ... | Downend, Gloucestershire |
| *Skerritt, E. Markham, M.D., B.S., B.A. Lond. | Richmond Hill, Clifton. |
| *Skinner, Stephen, M.B., C.M. Aberd. ... | Ferndale, Clevedon. |
| Smith, D. Boyes, M.D. Ed. ... ... ... ... | Eastcliff, Woolstone, Southampton. |
| *Smith, G. Munro ... ... ... ... ... ... ... | 70 Pembroke Road, Clifton. |
| *Smith, J. Greig, M.B., C.M., M.A. Ab., F.R.S.E. | 16 Victoria Square, Clifton. |
| Smith, J. Sidney, M.D. St. And. ... ... ... | Tiverton. |
| *Smith, R. Shingleton, M.D., B.Sc. Lond. | 9 Richmond Hill, Clifton. |
| Smith, Samuel ... ... ... ... ... ... ... | 7 Kingsdown Parade, Bristol. |
| Society, Cardiff Medical (*Hon. Sec.*, C. E. Hardyman). | |
| Society, Plymouth Medical (*Hon. Sec.*, C. Whipple). | |
| Society, Royal Medical and Chirurgical | 53 Berners Street, London, W. |
| Somer, James ... ... ... ... ... ... ... ... | Broadclyst. |
| *Spencer, W. H., M.D., M.A. Cantab. ... ... | 11 Warrior Square, St. Leonard's-on-Sea. |
| Spender, John K., M.D. Lond. ... ... ... | 17 Circus, Bath. |
| Stansfeld, G. M. ... ... ... ... ... ... ... | 19 Victoria Square, Clifton. |
| Statham, R. Whiteside ... ... ... ... ... | Cheddar, Somersetshire |
| Steel, Arthur R.... ... ... ... ... ... ... | Beer Alston, Devonshire. |
| Steel, S. H., M.B. Lond. ... ... ... ... ... | Abergavenny. |
| *Steele, Charles, M.D. Dur. ... ... ... ... | 17 Richmond Hill, Clifton. |
| Steele-Perkins, E. B. ... ... ... ... ... | 1 Hill's Buildings, St. Sidwell's, Exeter. |
| Stewart, James, B.A. Qu. Univ. I. ... ... | Dunmurry, Stoke Bishop. |
| Stockwell, Fred., M.D. Lond. ... ... ... | Bruton, Somersetshire. |
| Stoddart, F. Wallis ... ... ... ... ... ... | 1 Park Street, Bristol. |
| Storry, Frederick W. ... ... ... ... ... | Lansdown, Stroud. |
| *Swain, James, M.B., B.S. Lond. ... ... ... | Royal Infirmary, Bristol. |
| *Swayne, J. G., M.D. Lond. ... ... ... ... | 74 Pembroke Road, Clifton. |
| *Swayne, S. H.... ... ... ... ... ... ... ... | 129 Pembroke Road, Clifton. |
| Sydenham, George F. ... ... ... ... ... | Brushford, Exbridge. |
| | |
| Tait, Lawson ... ... ... ... ... ... ... ... | 7 The Crescent, Birmingham |
| *Taylor, James... ... ... ... ... ... ... ... | 36 Alma Road, Clifton. |
| Taylor, Thomas Henry ... ... ... ... ... | Thornbury, Gloucestershire. |
| Thomas, A. Garrod, M.D., C.M. Ed. ... ... | Clytha Park, Newport, Mon. |
| Thomas, G. Danford, M.D. Brussels ... ... | Park Lodge, Paddington, London, W. |
| Thomas, Jabez ... ... ... ... ... ... ... | Ty Cerrig, Swansea. |
| Thomas, R. W. ... ... ... ... ... ... ... | Freemantle Square, Bristol. |
| Thompson, George, M.D. Dur. ... ... ... | Asylum, Stapleton, Gloucestershire. |
| Thomson, G. S., M.D. Ed. ... ... ... ... | 8 College Road, Clifton. |
| Thurston, Hugh C. ... ... ... ... ... ... | 6 Whitehall Yard, London, S.W. |
| *Tivy, W. J. ... ... ... ... ... ... ... ... | 8 Lansdown Place, Clifton. |
| Todd, W. J. ... ... ... ... ... ... ... ... | North Petherton. |
| Tomkins, Harding H.... ... ... ... ... ... | 59 West Gate, Mansfield. |
| Torbock, W. H., M.D. Pennsylvania ... ... | Polruan, Cornwall. |
| Tosswill, Louis H., M.B., B.A. Cantab. ... | 28 Southernhay, Exeter. |
| Trask, J. E. ... ... ... ... ... ... ... ... | 6 Whitehall Yard, London, S.W. |

### LIST OF SUBSCRIBERS.

| | |
|---|---|
| Trotter, Leslie B., M.D., C.M. Ed. ... ... | Coleford, Gloucestershire. |
| Twining, A. H. ... ... ... ... ... ... ... | The Knoll, Salcombe. |
| Tyley, R. P., M.D. St. And. ... ... ... ... | Wedmore. |
| | |
| Urwick, W. ... ... ... ... ... ... ... ... | New Quay, Cornwall. |
| | |
| Vachell, C. T., M.D. Lond. ... ... ... ... | 38 Charles Street, Cardiff. |
| Vachell, Herbert R., M.D., C.M. Aberd. ... | 15 Newport Road, Cardiff. |
| Vernon, Edward ... ... ... ... ... ... ... | Kingston, Yeovil. |
| | |
| Wade, A. Law, M.D., B.A. Dub. ... ... ... | County Asylum, Wells, Somersetshire. |
| *Waldo, Henry, M.D., C.M. Aberd. ... ... | 19 Pembroke Road, Clifton. |
| Wallace, Rev. C. Hill, M.A. Oxon. ... ... | 3 Harley Place, Clifton. |
| Walter, William Henry, M.D. Brussels ... | Knap Villa, South Petherton. |
| Ward, J. L. W. ... ... ... ... ... ... ... | Victoria Street, Merthyr Tydvil. |
| *Warner, E. H., M.D., C.M. Ed. ... ... ... | Barton Hill House, Bristol. |
| *Wathen, J. Hancocke ... ... ... ... ... | 16 York Place, Clifton. |
| Watson, J. Adam ... ... ... ... ... ... ... | Chudleigh. |
| Watters, George T. B., M.D., C.M. Ed. ... | Stonehouse, Gloucestershire. |
| Waugh, Alexander ... ... ... ... ... ... | Midsomer Norton. |
| *Weatherly, Lionel A., M.D., C.M. Aberd. | Bailbrook House, Bath. |
| Webb, W. H. ... ... ... ... ... ... ... ... | Kingsbridge. |
| *Webster, Thomas ... ... ... ... ... ... | Redland Hill, Bristol. |
| *Welsh, F. F. ... ... ... ... ... ... ... ... | Rockleaze Point, Stoke Bishop. |
| Whiteley, George W. ... ... ... ... ... | Hamilton House, Downton, Wiltshire. |
| Whitfeld, W. Clarke ... ... ... ... ... | 5 St. Ethelbert Street, Hereford. |
| Whyte, Andrew, M.D., C.M. Aberd. ... ... | Honddû House, Brecon. |
| Wickham, W. ... ... ... ... ... ... ... ... | Hill House, Tetbury. |
| Wicksteed, Francis W. S. ... ... ... ... | The Villa Rosa, Weston-super-Mare. |
| Wigmore, J., M.D. Qu. Univ. I.... ... ... ... | Twerton-on-Avon. |
| *Wilding, J., M.B. Dur. ... ... ... ... ... | Foresters' Dispensary, Bristol. |
| Willcox, R. Lewis ... ... ... ... ... ... | Warminster. |
| Willett, George G. D. ... ... ... ... ... | 18 King Square, Bristol. |
| Williams, Charles ... ... ... ... ... ... | Port Isaac. |
| Williams, D. J. ... ... ... ... ... ... ... | Greenfield House, Llanelly. |
| Williams, H. E. ... ... ... ... ... ... ... | The Mount, Newport, Mon. |
| *Williams, P. Watson, M.B. Lond. ... ... | Royal Infirmary, Bristol. |
| Williams, Peter ... ... ... ... ... ... ... | Ferryside, Carmarthenshire. |
| Williams, William Henry, M.D. Lond. ... | Sherborne. |
| Wilson, Claude, M.D., C.M. Ed. ... ... ... | 6 York Road, Tunbridge Wells. |
| Wilton, John P. ... ... ... ... ... ... ... | 10 College Green, Gloucester. |
| Winterbotham, L. ... ... ... ... ... ... | Bays Hill, Cheltenham. |
| Wood, E. Stanley ... ... ... ... ... ... | Clarence House, Pontypool. |
| Woodd, H. T. ... ... ... ... ... ... ... ... | Calstock. |
| | |
| Yelf, Leonard K., M.D. St. And. ... ... ... | Moreton-in-Marsh. |

### *PUBLICATIONS RECEIVED.*

From THE WESTERN MEDICAL REPORTER, Chicago:
*Secondary Mixed Infection in Typhoid Fever.* By BAYARD HOLMES, M.D. Reprint. 1888.

From 95 WILLIAM STREET, New York:
*The International Journal of Surgery and Antiseptics.* Vol I. No. 2. April, 1888.

From THE AUTHOR, Philadelphia:
*A Case of Coloboma of the Iris, Lens and Choroid: with a Study of the Visual Fields.* By CHARLES A. OLIVER, M.D. Reprint. 1887.

From P. BLAKISTON, SON & CO., Philadelphia:
*The Polyclinic.* September, November, 1888.

From WM. J. DORNAN, Philadelphia:
*Report on Hydrophobia.* By CHARLES W. DULLES, M.D. Reprint from the Transactions of the Medical Society of the State of Pennsylvania. 1888.

From GOVERNMENT PRINTING OFFICE, Washington:
*Index-Catalogue of the Library of the Surgeon-General's Office, United States Army.* Vol. IX., MEDICINE (Popular)—NYWELT. 1888.

From J. W. ARROWSMITH, Bristol:
*On the Vomiting of Pregnancy and Menorrhagia.* By JOHN MEREDITH, M.D. 1888.

From THE AUTHORESS, London:
*Stirpiculture; or, The Scientific Propagation of the Human Race.* By VICTORIA CLAFLIN WOODHULL MARTIN. 1888.

From J. & A. CHURCHILL, London:
*Massage and Allied Methods of Treatment.* By HERBERT TIBBITS, M.D. Second Edition. 1888.
*Prevention of Disease in Tropical Campaigns.* By ANDREW DUNCAN, M.D., B.S. 1888.
"*B.P.C.*" *Unofficial Formulary.* 1888.
*Puerperal Fever.* By CHARLES J. CULLINGWORTH, M.D. 1888.
*Alpine Winter in its Medical Aspects.* By A. TUCKER WISE, M.D. Fourth Edition. 1888.
*On the Treatment of Cystic Goître.* By T. MARK HOVELL. 1888.
*On Tension, Inflammation of Bone, and Head Injuries.* By THOMAS BRYANT. 1888.
*Diseases of the Urinary Organs.* By SIR HENRY THOMPSON. Eighth Edition. 1888.

From H. K. LEWIS, London:
*Elements of Practical Medicine.* By ALFRED H. CARTER, M.D. Fifth Edition. 1888.
*A Manual of Ophthalmic Practice.* By CHARLES HIGGENS. 1888.
*Hydrophobia.* By W. COLLIER, M.D. 1888.
*The British Journal of Dermatology.* Vol. I. No. 1. November, 1888.
*Epitome of Ear Diseases.* By W. R. H. STEWART. 1888.
*Section Cutting and Staining.* By WALTER S. COLMAN, M.B. 1888.
*Surface Anatomy.* By BERTRAM C. A. WINDLE, M.A., M.D. 1888.
*Ambulance Work.* By R. LAWTON ROBERTS, M.D. Third Edition. 1888.
*Handbook of Therapeutics.* By SYDNEY RINGER, M.D., F.R.S. Twelfth Edition. 1888.
*Displacements of the Uterus.* By Dr. B. S. SCHULTZE. 1888.

From JOHN HEYWOOD, Manchester:
*The Treatment of Empyema.* By G. J. ROBERTSON, M.B., C.M. 1888.

From J. YOUNG & SONS, Perth:
*The Sixty-first Annual Report of James Murray's Royal Asylum, Perth.* 1888.

*Cases for binding, price 7d. each, may be had of the Publisher,*
J. W. ARROWSMITH, *Quay Street, Bristol.*

